# COMMD3 coordinates mannose-6-phosphate receptor trafficking to sustain lysosomal protease maturation

**DOI:** 10.21203/rs.3.rs-10821157/v1

**Published:** 2026-09-15

**Authors:** Yixiang Hu, Yong-Hui Zheng

**Affiliations:** Department of Microbiology and Immunology, The University of Illinois; Chicago, IL 60612, USA.

**Keywords:** COMMD3, CI-MPR, CD-MPR, Cathepsins, Endosomes, virus entry, SARS-CoV-2, Ebola

## Abstract

Lysosomal function depends on the cation-independent and cation-dependent mannose-6-phosphate receptors (CI-MPR and CD-MPR), which deliver newly synthesized acid hydrolases from the trans-Golgi network (TGN) to the endolysosomal system. However, how the trafficking of these receptors is coordinately regulated remains incompletely understood. Here we identify COMMD3 as a regulator of both MPRs that promotes their exit from early endosomes and sustains lysosomal hydrolase maturation. Loss of COMMD3 causes accumulation of CI-MPR and CD-MPR in early endosomal compartments and profoundly impairs the maturation and activity of Cathepsin L (CTSL). Combined deletion of both MPRs phenocopies COMMD3 deficiency, functionally linking COMMD3-dependent MPR trafficking to CTSL maturation. Unexpectedly, this trafficking activity is mediated primarily by the N-terminal domain of COMMD3 rather than its canonical C-terminal COMM domain involved in Commander complex assembly. Disruption of COMMD3 or both MPRs also selectively restricts CTSL-dependent entry of SARS-CoV-2 and Ebola virus. Together, our findings uncover a COMMD3-MPR trafficking axis that couples endosomal sorting to lysosomal protease maturation and supports endosome-dependent viral entry.

## Introduction

Lysosomes are essential degradative organelles that maintain cellular homeostasis through the turnover of proteins, lipids, pathogens and damaged organelles^1^. Their function depends on the delivery of newly synthesized acid hydrolases from the trans-Golgi network (TGN) to endosomal compartments, a process mediated primarily by the cation-independent and cation-dependent mannose-6-phosphate receptors (CI-MPR and CD-MPR)^2^. Disruption of lysosomal hydrolase trafficking compromises lysosomal function and contributes to diverse human diseases, including lysosomal storage disorders and neurodegenerative diseases^1^. Among these hydrolases, Cathepsin L (CTSL) is a major lysosomal cysteine protease that undergoes proteolytic maturation following delivery to acidic endolysosomal compartments^1, 2^. In addition to its role in cellular proteolysis, CTSL is exploited by several endosome-dependent viruses, including severe acute respiratory syndrome coronavirus 2 (SARS-CoV-2) and Ebola virus (EBOV), to proteolytically activate their surface glycoproteins for membrane fusion and entry^3^. Thus, mechanisms controlling CTSL trafficking and maturation have important consequences for both lysosomal homeostasis and viral infection.

CI-MPR and CD-MPR capture mannose-6-phosphate-modified hydrolases at the TGN and transport them in clathrin-coated carriers to endosomes, where acidic pH promotes cargo release. The released hydrolases subsequently progress to lysosomes and undergo proteolytic maturation, whereas MPRs undergo repeated rounds of intracellular sorting and recycling to sustain hydrolase delivery. MPR trafficking is controlled by an interconnected network of endosomal sorting machineries, including retromer-associated complexes^4^, sorting nexins^5^ and the WASH actin-polymerization machinery^6^, which coordinate cargo recognition, membrane remodeling and transport-carrier formation. Despite extensive characterization of these pathways, how MPRs are coordinated through the endosomal system to sustain lysosomal hydrolase delivery remains incompletely understood.

The Commander complex is an evolutionarily conserved endosomal trafficking machinery composed of 16 subunits, including ten COMMD proteins and the Retriever and CCC subcomplexes^7, 8^. Commander regulates the sorting and recycling of diverse transmembrane proteins and is required to maintain the cell-surface abundance of cargos including integrins and low-density lipoprotein receptors^9, 10^. COMMD proteins share a conserved C-terminal COMM domain that mediates their incorporation into the Commander complex, whereas their more divergent N-terminal regions remain less well characterized. Notably, recent studies have linked COMMD3 deficiency to lysosomal dysfunction and Parkinson’s disease risk^11^, suggesting an unexpected role for COMMD3 in lysosomal homeostasis. However, the underlying trafficking pathway and whether COMMD3 directly coordinates lysosomal enzyme delivery remain unknown.

Here, we identify COMMD3 as a critical regulator of MPR trafficking and lysosomal CTSL maturation. We show that COMMD3 associates with CD-MPR predominantly in early endosomes and promotes the progression of both CI-MPR and CD-MPR from early to later endosomal compartments. Loss of COMMD3 causes MPR accumulation in early endosomes, profoundly impairs CTSL maturation and activity, and selectively restricts CTSL-dependent entry of SARS-CoV-2 and EBOV. Genetic disruption of both MPRs phenocopies COMMD3 deficiency, establishing a functional link between COMMD3-dependent MPR trafficking, CTSL maturation and viral entry. Unexpectedly, this activity is mediated primarily by the N-terminal domain of COMMD3 rather than its canonical C-terminal COMM domain. Together, our findings uncover a previously unrecognized function of COMMD3 in coupling endosomal MPR trafficking to lysosomal protease maturation and reveal how this pathway supports endosome-dependent viral entry.

## Results

### COMMD3 is essential for SARS-CoV-2 endocytic and Ebola virus entry.

Four independent genome-wide CRISPR screens identified COMMD3 as a host factor required for SARS-CoV-2 infection^12–15^. However, its precise role remains unclear, as one study attributed the requirement for COMMD3 to regulation of the SARS-CoV-2 receptor ACE2 surface expression^15^, whereas another found no such effect^12^. To define the role of COMMD3 in SARS-CoV-2 infection, we disrupted *COMMD3* (*COM3*) in human A549 cells ectopically expressing ACE2 (A549-A cells), with gene disruption confirmed by genomic sequencing (**Fig. S1**, top panel).

**Fig. 1.**
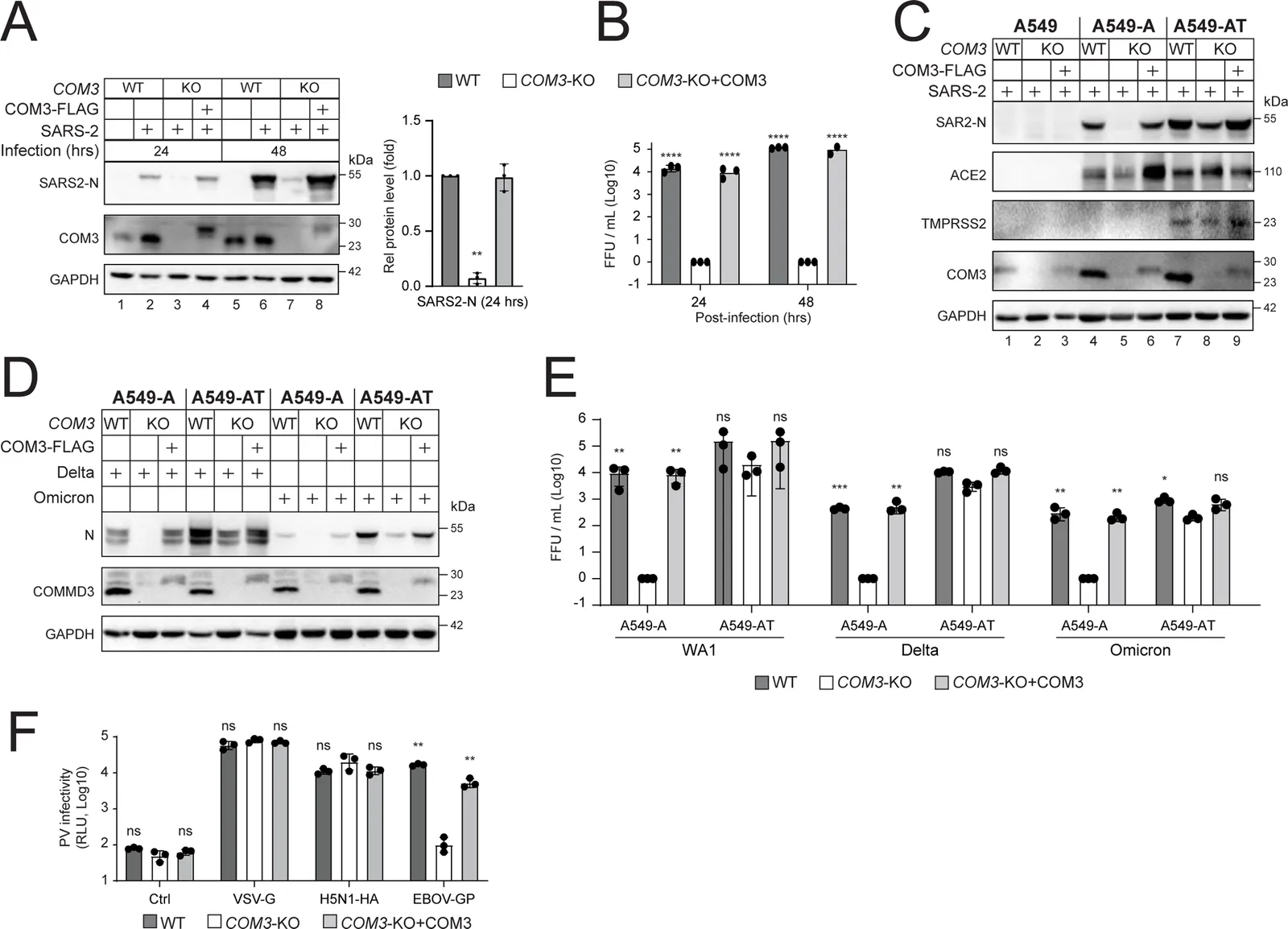
COMMD3 is essential for SARS-CoV-2 endocytic and Ebola virus entry. **A.** WT, COM3-KO, and rescued A549-A cells were infected with SARS-CoV-2 (SARS2) WA1 at an MOI of 0.1 and harvested at 24 or 48 h post-infection. Western blot analysis of SARS2 nucleocapsid (N) protein is shown. Densitometric quantification of N protein levels at 24 h post-infection is shown at right. N protein levels were normalized to GAPDH and expressed relative to WT. Data are presented as mean ± SD. Each dot represents one biologically independent experiment (*n* = 3). One-way ANOVA followed by Dunnett’s multiple-comparison test using WT as the control. ns, not significant; **P < 0.01. **B.** Viral titers in culture supernatants from WT, COM3-KO, and rescued A549-A cells were determined by immunostaining-based focus-forming unit (FFU) assay. Data are presented as mean ± SD. Each dot represents one biologically independent experiment (*n* = 3). Two-way ANOVA followed by Dunnett’s multiple-comparison test using COM3-KO as the control. ns, not significant; ****P < 0.0001. **C.** WT, COM3-KO, and rescued A549, A549-A, and A549-AT cells were infected with SARS2 WA1 strain at an MOI of 0.1 and harvested at 24, followed by western blot validation of SARS2 N protein, ACE2, and TMPRSS2 expression. **D.** WT, COM3-KO, and rescued A549-A and A549-AT cells were infected with SARS2 Delta or Omicron strain at an MOI of 0.1 and harvested at 24, followed by western blot validation of SARS2 N protein expression. **E.** WT, COM3-KO, rescued A549-A, and TMPRSS2-overexpressing A549-A (A549-AT) cells were infected with SARS2 WA1, Delta, or Omicron at an MOI of 0.1 and harvested at 24 h post-infection. Viral titers in culture supernatants were determined by FFU. Data are presented as mean ± SD. Each dot represents one biologically independent experiment (*n* = 3). Two-way ANOVA followed by Dunnett’s multiple-comparison test using the COM3-KO group as the control. ns, not significant; *P < 0.05; **P < 0.01; ***P < 0.001. **F.** WT, COM3-KO, and rescued A549-A cells were infected with HIV-1-based lentiviral pseudoviruses (PVs) bearing the indicated viral glycoproteins. Firefly luciferase activity was measured at 48 h post-infection. Data are presented as mean ± SD. Each dot represents one biologically independent experiment (*n* = 3). Two-way ANOVA followed by Dunnett’s multiple-comparison test using the COM3-KO group as the control. ns, not significant; *P < 0.05; **P < 0.01.

We first examined infection with the ancestral SARS-CoV-2 WA1 strain. At 24 h post-infection (hpi), viral nucleocapsid (N) protein expression was reduced 13.5-fold in *COM3*-knockout (KO) cells compared with wild-type (WT) cells, and much more profound reduction was observed at 48 hpi (P = 0.0015; Fig. 1A). Consistent with this reduction, focus-forming unit (FFU) assays showed that production of infectious progeny virus was nearly abolished in *COM3*-KO cells at both 24 and 48 hpi (P < 0.0001 for both time points; Fig. 1B). Re-expression of COMMD3 fully restored viral protein expression (Fig. 1A, lanes 4 and 8) and infectious virus production (Fig. 1B), demonstrating that the phenotype resulted specifically from COMMD3 deficiency. Similar results were obtained with a GFP-expressing SARS-CoV-2 reporter virus (**Fig. S2A**) and an independent *COM3*-KO clone (**Fig. S2B**).

SARS-CoV-2 (SARS-2) can enter cells through either endosomal entry or TMPRSS2-dependent fusion at the plasma membrane. We therefore expressed TMPRSS2 in WT and *COM3*-KO A549-A cells to generate A549-AT cells. COMMD3 deficiency markedly impaired infection by the WA1, Delta and Omicron strains in A549-A cells, whereas expression of TMPRSS2 largely rescued this defect, as determined by immunoblotting (**Fig. 1C-D**) and FFU assays (Fig. 1E). A similar TMPRSS2-dependent rescue was observed in *COM3*-KO HEK293T cells (**Fig. S2C**). These findings suggested that COMMD3 is specifically required for the endosomal route of SARS-CoV-2 entry.

**Fig. 2.**
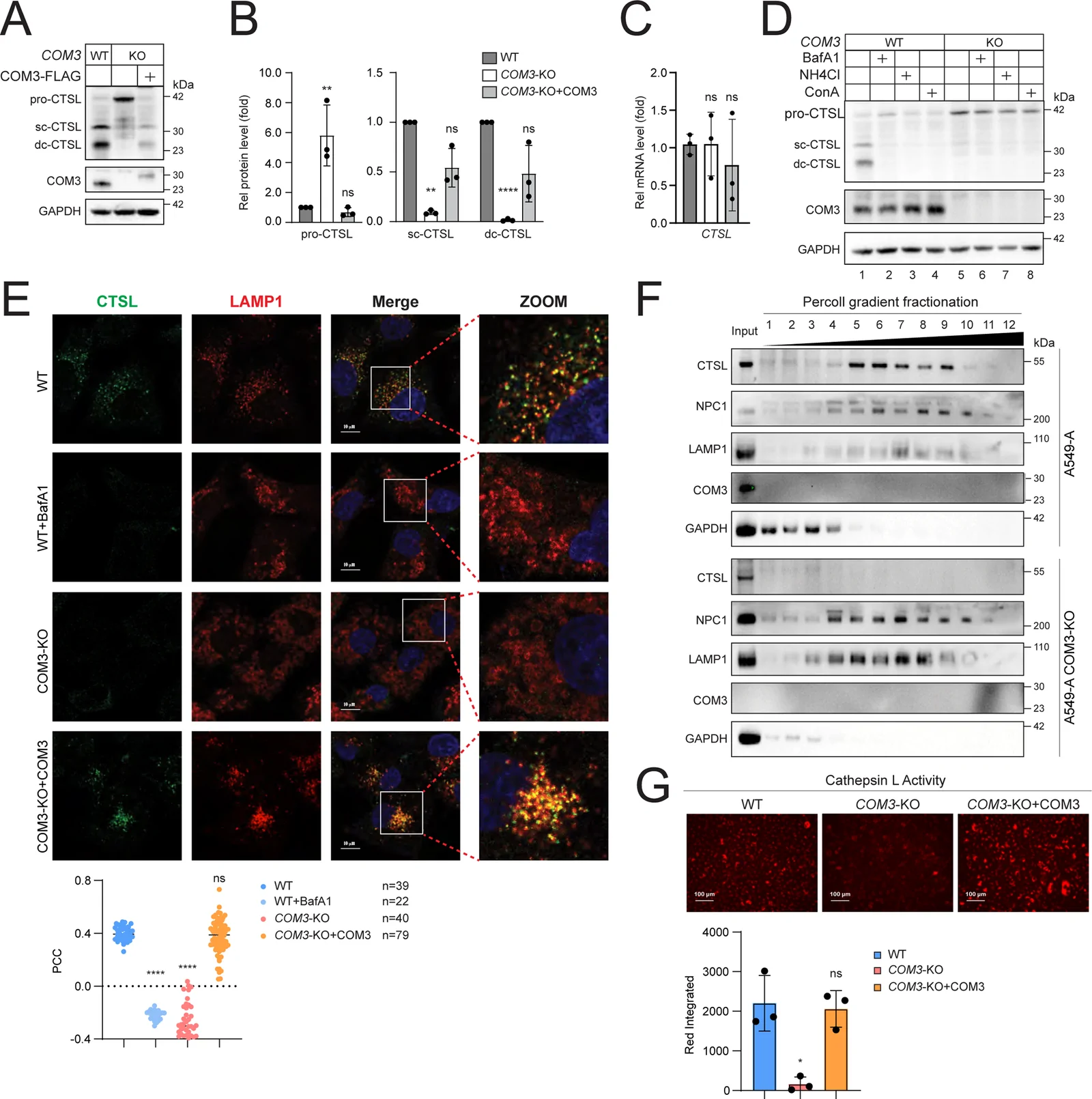
COMMD3 sustains Cathepsin L maturation. **A.** Western blot analysis of CTSL expression in WT, COM3-KO, and rescued A549-A cells. **B.** Densitometric quantification of pro-CTSL, single-chain CTSL (sc-CTSL), and double-chain CTSL (dc-CTSL) levels in WT, COM3-KO, and rescued A549-A cells. Pro-CTSL levels were normalized to GAPDH and expressed relative to WT. Sc-CTSL and dc-CTSL levels were normalized to pro-CTSL levels and expressed relative to WT. Data are presented as mean ± SD. Each dot represents one biologically independent experiment (*n* = 3). One-way ANOVA followed by Dunnett’s multiple-comparison test using WT as the control. ns, not significant; **P < 0.01; ****P < 0.0001. **C.** RT-qPCR analysis of CTSL mRNA levels in WT, COM3-KO, and rescued A549-A cells. Data are presented as mean ± SD. Each dot represents one biologically independent experiment (*n* = 3). One-way ANOVA followed by Dunnett’s multiple-comparison test using WT as the control. **D.** WT and COM3-KO A549-A cells were treated with 100 nM bafilomycin A1 (BafA1), 20 mM ammonium chloride (NH_4_Cl), or 100 nM concanamycin A (ConA) for 12 h, followed by western blot analysis of CTSL expression. **E.** Representative immunofluorescence images of WT, BafA1-treated WT, COM3-KO, and rescued A549-A cells. Green, CTSL; red, LAMP1; blue, DAPI. Quantification of Pearson’s correlation coefficient (PCC) between CTSL and LAMP1 in cells is shown below the images. PCC was calculated using an intensity threshold of 0.05. Each dot represents one field of view. Sample sizes are indicated in the figure. One-way ANOVA followed by Dunnett’s multiple-comparison test using WT as the control. ns, not significant; ****P < 0.0001. **F.** Western blot analysis of the indicated proteins in Percoll gradient fractions from WT and COM3-KO A549-A cells. Twelve fractions were collected from the top (fraction 1) to the bottom (fraction 12) of the gradient. **G.** Representative fluorescence images showing CTSL activity in WT, COM3-KO, and rescued A549-A cells. Cells were incubated with the Magic Red CTSL substrate for 30 min before imaging. Quantification of integrated Magic Red fluorescence intensity in the cells is shown below the images. Integrated fluorescence intensity was measured from pixels above a fixed threshold (80–255) applied identically to all images. Data are presented as mean ± SD. Each dot represents one biologically independent experiment (*n* = 3). One-way ANOVA followed by Dunnett’s multiple-comparison test using WT as the control. ns, not significant; *P < 0.05.

We next asked whether the entry defect resulted from altered expression of the viral receptor ACE2 or TMPRSS2. COMMD3 deficiency did not reduce total ACE2 abundance in either A549-A or A549-AT cells (Fig. 1C, lanes 4–5 and 7–8), and flow cytometry similarly revealed no reduction in cell-surface ACE2 (**Fig. S3A**). TMPRSS2 expression was also unaffected by COMMD3 deficiency (Fig. 1C, lanes 7–8). Moreover, loss of COMMD3 did not impair SARS-CoV-2 Spike-mediated cell-cell fusion (**Fig. S3B**). Furthermore, loss of COMMD3 did not reduce NPC1 expression (Fig. 2C), which is required for SARS-CoV-2 endocytic entry^16, 17^. Thus, the requirement for COMMD3 cannot be explained by reduced ACE2 and NPC1 expression, impaired ACE2 surface localization or a general defect in Spike-mediated membrane fusion.

Together, these results identify COMMD3 as a selective requirement for endosomal SARS-CoV-2 entry while being dispensable for TMPRSS2-mediated entry at the plasma membrane.

We next examined whether this requirement extends to other viruses that enter through endocytic pathways. HIV-1 luciferase-reporter pseudovirions (PVs) were pseudotyped with Ebola virus (EBOV) glycoprotein (GP), vesicular stomatitis virus (VSV) glycoprotein G or influenza A virus (IAV; H5N1) hemagglutinin (HA) and neuraminidase (NA). COMMD3 deficiency reduced EBOV-GP-mediated entry by more than 2 log10 (P = 0.0053), which was almost fully restored by COMMD3 re-expression, whereas VSV-G- and H5N1-mediated entry was unaffected (Fig. 1F). Thus, COMMD3 is not broadly required for endocytic viral entry but is selectively required by SARS-CoV-2 and EBOV.

### COMMD3 sustains Cathepsin L maturation.

Endosomal entry of SARS-CoV-2 and EBOV shares a requirement for cathepsin L (CTSL), a major lysosomal cysteine protease that proteolytically primes their viral glycoproteins for membrane fusion in late endosomal compartments. CTSL is synthesized as an inactive precursor (pro-CTSL) and undergoes sequential proteolytic processing in acidic endolysosomal compartments to generate the mature single-chain (sc-CTSL) and double-chain (dc-CTSL) forms. We therefore investigated whether COMMD3 regulates CTSL maturation.

Immunoblot analysis revealed a pronounced defect in CTSL processing in *COM3*-KO cells (**Fig. 2A-B**). Pro-CTSL accumulated 5.8-fold relative to WT cells (P = 0.0100), whereas mature sc-CTSL and dc-CTSL were reduced by 10.9-fold (P = 0.0003) and 69.8-fold (P < 0.0001), respectively. Re-expression of COMMD3 restored both mature CTSL species, demonstrating that the defect was specifically attributable to COMMD3 loss.

CTSL transcript abundance was unchanged following COMMD3 deletion (Fig. 2C), indicating that the altered CTSL profile arose post-transcriptionally. We next asked whether accumulation of pro-CTSL could be explained solely by impaired proteolytic maturation resulting from defective lysosomal acidification. WT cells were treated with bafilomycin A1, ammonium chloride or concanamycin A to disrupt endolysosomal acidification. Although each treatment impaired CTSL maturation, none recapitulated the pronounced accumulation of pro-CTSL observed in *COM3*-KO cells (Fig. 2D). These findings suggested that COMMD3 deficiency affects CTSL through a mechanism beyond inhibition of its acidification-dependent proteolytic processing.

We next examined CTSL localization by confocal microscopy. In WT cells, CTSL displayed a punctate intracellular distribution and colocalized with the lysosomal marker LAMP1 (Fig. 2E). CTSL staining was abolished following bafilomycin A1 treatment, despite the ability of the antibody to recognize both precursor and mature CTSL by immunoblotting, indicating that under these immunofluorescence conditions the signal predominantly represents mature CTSL. Consistent with the biochemical defect, CTSL staining was nearly undetectable in *COM3*-KO cells but was restored following COMMD3 re-expression, where it again colocalized with LAMP1 (Fig. 2E). Similar results were obtained using the late-endosomal marker RAB7A (**Fig. S4**), further supporting a requirement for COMMD3 in generating mature CTSL within endolysosomal compartments.

To independently assess the subcellular distribution of CTSL, we performed Percoll density-gradient fractionation. In WT cells, mature CTSL predominantly cofractionated with the lysosomal markers NPC1 and LAMP1. By contrast, mature CTSL was largely absent from these Percoll fractions derived from *COM3*-KO cells (Fig. 2F), confirming a marked loss of mature CTSL from lysosomal compartments.

Consistent with these biochemical and imaging results, intracellular CTSL enzymatic activity was reduced 13.6-fold following COMMD3 deletion (P = 0.0010) and was fully restored by COMMD3 re-expression (Fig. 2G). By contrast, the previously described MAN1B1-dependent lysosomal degradation pathway^18^ remained intact in *COM3*-KO cells (**Fig. S5**), indicating that COMMD3 deficiency does not cause a general loss of lysosomal degradative capacity.

Together, these results establish COMMD3 as a critical determinant of CTSL maturation and activity and suggest that its loss disrupts a specific pathway required for the generation of functional CTSL within endolysosomal compartments.

### COMMD3 deficiency disrupts endolysosomal homeostasis and causes MPR accumulation.

**Fig. 3.**
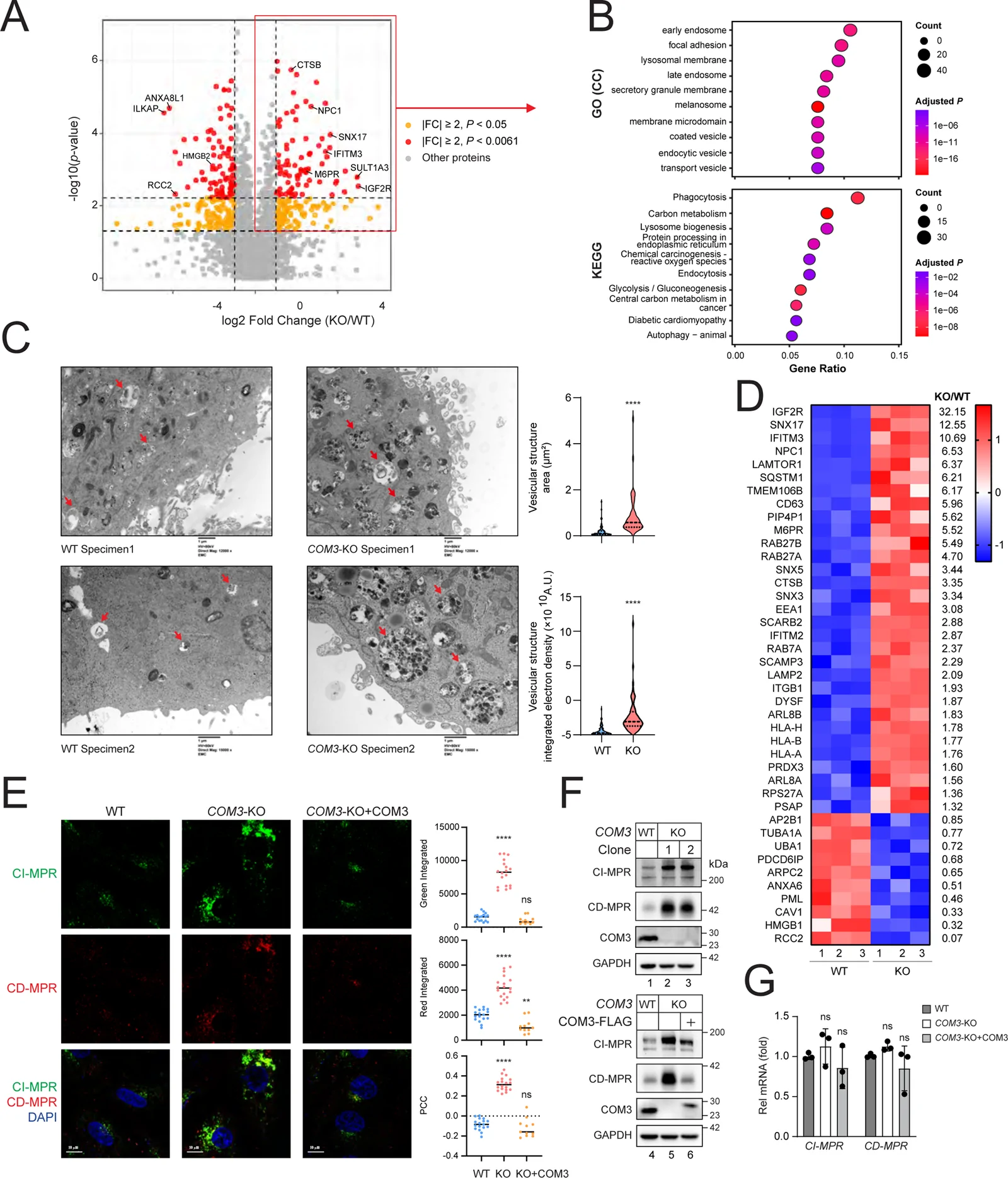
COMMD3 deficiency disrupts endolysosomal homeostasis and causes MPR accumulation. **A.** Volcano plot showing differential protein expression between WT and COMMD3-KO A549-A cells determined by quantitative mass spectrometry. Proteins with |FC| ≥ 2 and *P* < 0.05 are shown in orange, and those meeting the family-wise error rate (FWER)-corrected threshold (|FC| ≥ 2, *P*< 0.0061) are shown in red. All other proteins are shown in gray. **B.** Gene Ontology (GO) Cellular Component (CC) and Kyoto Encyclopedia of Genes and Genomes (KEGG) pathway enrichment analyses of proteins significantly upregulated in COM3-KO cells relative to WT (*P* < 0.05). **C.** Transmission electron microscopy (TEM) images of WT and COM3-KO A549-A cells acquired at ×12,000 and ×15,000 magnification. Representative vesicular structures are indicated by arrows. Quantification of vesicular structure area and integrated electron density is shown at right (WT, *n* = 54; KO, *n* = 50). Welch’s *t* test. ****P < 0.0001. **D.** Z score–normalized heatmap of the proteins shown in Fig. S6B. **E.** Representative immunofluorescence images of CI-MPR (green), CD-MPR (red), and nuclei (DAPI, blue) in WT, COM3-KO, and rescued A549-A cells. Quantification of CI-MPR and CD-MPR fluorescence intensities and Pearson’s correlation coefficient (PCC) is shown at right. Measurements were performed using an intensity threshold of 0.05. Each dot represents one field of view (WT, *n* = 17; COM3-KO, *n* = 18; COM3-KO+COM3, *n* = 11). One-way ANOVA followed by Dunnett’s multiple-comparison test using WT as the control. ns, not significant; **P < 0.01; ****P < 0.0001. **F.** Western blot analysis of CI-MPR and CD-MPR expression in A549-A cells. Top, WT and two independent COMMD3-KO clones. Bottom, WT, COM3-KO clone 1, and rescued COM3-KO clone 1. **G.** RT-qPCR analysis of CI-MPR and CD-MPR mRNA levels in WT, COM3-KO, and rescued A549-A cells. Data are presented as mean ± SD. Each dot represents one biologically independent experiment (*n* = 3). One-way ANOVA followed by Dunnett’s multiple-comparison test using WT as the control. ns, not significant.

To investigate the mechanism underlying the effects of COMMD3 deficiency on viral entry and CTSL maturation, we quantitatively compared the proteomes of WT and *COM3*-KO A549-A cells by mass spectrometry (**Table S1**). COMMD3 deletion induced extensive proteomic remodeling, with 374 proteins significantly increased and 533 proteins significantly decreased (P < 0.05; Fig. 3A).

Functional enrichment analysis revealed that proteins increased in *COM3*-KO cells were strongly enriched in endolysosomal compartments, including early endosomes, late endosomes and lysosomal membranes, as well as pathways related to endocytosis, phagocytosis and lysosome biogenesis (Fig. 3B). By contrast, proteins decreased following COMMD3 deletion were predominantly associated with focal adhesion and IgSF CAM signaling pathways (**Fig. S6A**), consistent with the established role of the Commander complex in membrane protein recycling.

Transmission electron microscopy further revealed pronounced ultrastructural abnormalities following COMMD3 loss. Compared with WT cells, *COM3*-KO cells accumulated enlarged electron-dense organelles with significantly increased area and electron density (P < 0.0001 for both; Fig. 3C). Enlarged early endosomes have similarly been observed in COMMD3-deficient cells by confocal microscopy^19^. Together with the proteomic enrichment of endolysosomal proteins, these findings indicate that COMMD3 is required to maintain endolysosomal homeostasis.

To identify specific endosomal proteins affected by COMMD3 deficiency, we next focused on proteins annotated to the endosome (GO:0005768). Of 223 endosome-associated proteins detected, 41 remained significantly altered after family-wise error rate correction (P < 0.0061; **Fig. S6B**). Strikingly, the two mannose-6-phosphate receptors (MPRs) were among the most strongly affected proteins. CI-MPR (IGF2R) showed the largest increase of all endosomal proteins, increasing 32.2-fold (P = 0.0029), whereas CD-MPR (M6PR) increased 5.5-fold (P = 0.0011) (Fig. 3D). Because CI-MPR and CD-MPR cooperate in the delivery of newly synthesized lysosomal hydrolases from the TGN to endosomes, their coordinated accumulation suggested that COMMD3 may regulate a shared MPR trafficking pathway. Consistent with this interpretation, STRING interaction analysis placed CI-MPR and CD-MPR within a highly connected functional module (**Fig. S6C**).

Immunofluorescence microscopy confirmed marked intracellular accumulation of both CI-MPR and CD-MPR in *COM3*-KO cells, which was reversed by COMMD3 re-expression (P < 0.0001; Fig. 3E, top and middle panels). COMMD3 deletion also increased colocalization between the two receptors, an effect that was reversed by COMMD3 re-expression (Fig. 3E, bottom panel). Although the substantially stronger CI-MPR fluorescence partially obscured the weaker CD-MPR signal in the merged images, quantitative colocalization analysis revealed a marked increase in the Pearson correlation coefficient (ΔPCC = 0.404, P < 0.0001). These results indicate that CI-MPR and CD-MPR accumulate within common intracellular compartments following COMMD3 loss.

Immunoblotting independently confirmed increased CI-MPR and CD-MPR abundance in *COM3*-KO cells (Fig. 3F, lanes 1–3), whereas COMMD3 re-expression restored both receptors towards WT levels (lane 6). Similar MPR accumulation accompanied by impaired CTSL maturation was observed in A549 and A549-AT cells (**Fig. S7**), indicating that these effects were not restricted to A549-A cells. By contrast, CI-MPR and CD-MPR transcript abundance was unchanged following COMMD3 deletion (Fig. 3G), demonstrating that their accumulation occurs through a post-transcriptional mechanism.

Together, these results reveal extensive endolysosomal remodeling following COMMD3 loss and identify CI-MPR and CD-MPR as prominent proteins that accumulate in COMMD3-deficient cells. The coordinated accumulation of both receptors, together with the defect in CTSL maturation, raised the possibility that COMMD3 regulates a shared intracellular trafficking pathway required for MPR function. We therefore next investigated how COMMD3 controls the intracellular trafficking of CI-MPR and CD-MPR.

### COMMD3 promotes MPR exit from early endosomes.

**Fig. 4.**
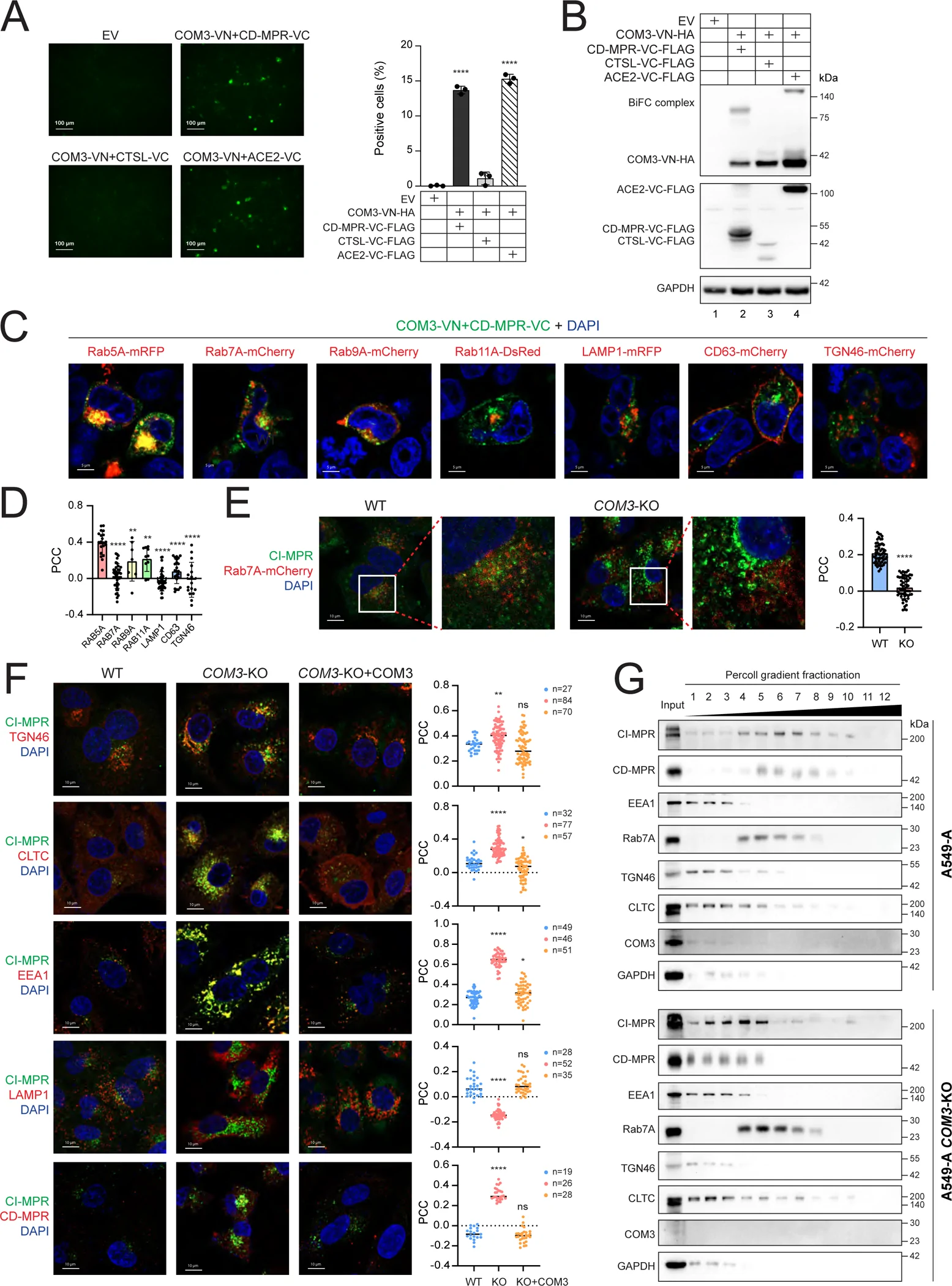
COMMD3 promotes MPR exit from early endosomes. **A.** Representative fluorescence images of HEK293T cells transfected with the indicated constructs for 36 h are shown at left. Quantification of BiFC-positive cells by flow cytometry is shown at right. Data are presented as mean ± SD. Each dot represents one biologically independent experiment (*n* = 3). One-way ANOVA followed by Dunnett’s multiple-comparison test using WT as the control. ns, not significant; ****P < 0.0001. EV, empty vectors. **B.** Western blot analysis of the indicated BiFC fusion proteins in transfected HEK293T cells. **C.** Representative confocal images of HEK293T cells transfected with the indicated constructs for 36 h. Green, BiFC signal; red, mRFP or mCherry-tagged organelle markers; blue, DAPI. **D.** Quantification of Pearson’s correlation coefficient (PCC) between the BiFC signal and the indicated organelle markers. PCC was calculated using an intensity threshold of 0.05. Data are presented as mean ± SD. Each dot represents one cell positive for both BiFC and mCherry fluorescence (RAB5A, *n* = 20; RAB7A, *n* = 37; RAB9A, *n* = 7; RAB11A, *n* = 10; LAMP1, *n* = 39; CD63, *n* = 23; TGN46, *n* = 23). One-way ANOVA followed by Dunnett’s multiple-comparison test using the RAB5A group as the control. **P < 0.01; ****P < 0.0001. **E.** Representative immunofluorescence images of WT and COM3-KO A549-A cells stably expressing RAB7A-mCherry. Green, CI-MPR; red, RAB7A-mCherry; blue, DAPI. Quantification of PCC between CI-MPR and RAB7A-mCherry is shown at right. PCC was calculated using an intensity threshold of 0.05. Data are presented as mean ± SD. Each dot represents one field of view (WT, *n* = 57; COM3-KO, *n* = 57). Welch’s *t* test. ****P < 0.0001. **F.** Representative immunofluorescence images of WT, COMMD3-KO, and rescued A549-A cells. Green, CI-MPR; red, the indicated organelle markers; blue, DAPI. Quantification of PCC between CI-MPR and the indicated organelle markers is shown at right. PCC was calculated using an intensity threshold of 0.05. Each dot represents one field of view. Sample sizes are indicated in the figure. One-way ANOVA followed by Dunnett’s multiple-comparison test using WT as the control. ns, not significant; *P < 0.05; **P < 0.01; ****P < 0.0001. **G.** Western blot analysis of the indicated proteins in Percoll gradient fractions from WT and COM3-KO A549-A cells. Twelve fractions were collected from the top (fraction 1) to the bottom (fraction 12) of the gradient.

To determine whether COMMD3 associates with MPRs, we performed bimolecular fluorescence complementation (BiFC), in which interaction-dependent proximity reconstitutes the N-terminal (VN173) and C-terminal (VC155) fragments of Venus fluorescent protein. COMMD3 was fused to VN173, whereas CD-MPR, CTSL or ACE2 was individually fused to VC155. Following co-expression in HEK293T cells, robust BiFC fluorescence was detected between COMMD3 and CD-MPR, comparable to that observed with the positive control ACE2, whereas no detectable signal was observed with CTSL (Fig. 4A). Flow cytometric analysis showed that 13.7 ± 0.6% and 15.3 ± 0.7% of cells were Venus-positive in the COMMD3-CD-MPR and COMMD3-ACE2 groups, respectively. Immunoblotting confirmed comparable expression of the fusion proteins and detected the characteristic stable BiFC complex (Fig. 4B), supporting an intracellular association between COMMD3 and CD-MPR.

We next determined the subcellular compartment in which this association occurs by co-expressing the COMMD3 and CD-MPR BiFC pair with fluorescently tagged organelle markers. The BiFC signal showed the strongest colocalization with the early endosome marker RAB5A (PCC = 0.392 ± 0.129), whereas substantially weaker overlap was observed with RAB9A and RAB11A and little colocalization was detected with RAB7A, LAMP1, CD63 or TGN46 (**Fig. 4C-D**). These results indicate that COMMD3 associates with CD-MPR predominantly within early endosomal compartments.

We therefore asked whether COMMD3 regulates MPR progression through the endosomal system. In WT A549-A cells, CI-MPR showed substantial colocalization with RAB7A-positive late endosomes. By contrast, COMMD3 deficiency markedly reduced CI-MPR colocalization with RAB7A (ΔPCC = 0.19, P < 0.0001; Fig. 4E), consistent with impaired trafficking from early to later endosomal compartments.

A broader analysis of CI-MPR localization further supported this defect. COMMD3 deletion significantly increased CI-MPR colocalization with another early endosome marker EEA1 (ΔPCC = 0.38, P < 0.0001) and the clathrin heavy-chain marker CLTC (ΔPCC = 0.18, P < 0.0001), while producing only a modest increase in colocalization with TGN46 (ΔPCC = 0.07, P = 0.0075). In contrast, CI-MPR colocalization with the late endosome/lysosome marker LAMP1 was markedly reduced (ΔPCC = 0.22, P < 0.0001). Re-expression of COMMD3 restored the intracellular distribution of CI-MPR towards that observed in WT cells (Fig. 4F). Together, these findings indicate that loss of COMMD3 causes CI-MPR to accumulate in early endosomal and clathrin-associated compartments while reducing its progression to later endolysosomal compartments.

To independently validate these imaging results, we separated intracellular membranes by Percoll density-gradient centrifugation. In WT cells, CI-MPR and CD-MPR predominantly cofractionated with the late endosome marker RAB7A in intermediate-density fractions. In *COM3*-KO cells, however, both receptors shifted towards lighter fractions enriched for the early endosome marker EEA1 (Fig. 4G). Thus, loss of COMMD3 redistributes both MPRs from late endosomal towards early endosomal compartments.

Together, these results identify COMMD3 as a regulator of MPR trafficking from early endosomes. COMMD3 associates with CD-MPR primarily in early endosomal compartments, and its loss causes both CI-MPR and CD-MPR to accumulate in early endosomes at the expense of later endosomal localization. We therefore next asked whether impaired MPR trafficking compromises the delivery and maturation of lysosomal hydrolases.

### MPR-dependent trafficking links COMMD3 to CTSL maturation and viral entry.

**Fig. 5.**
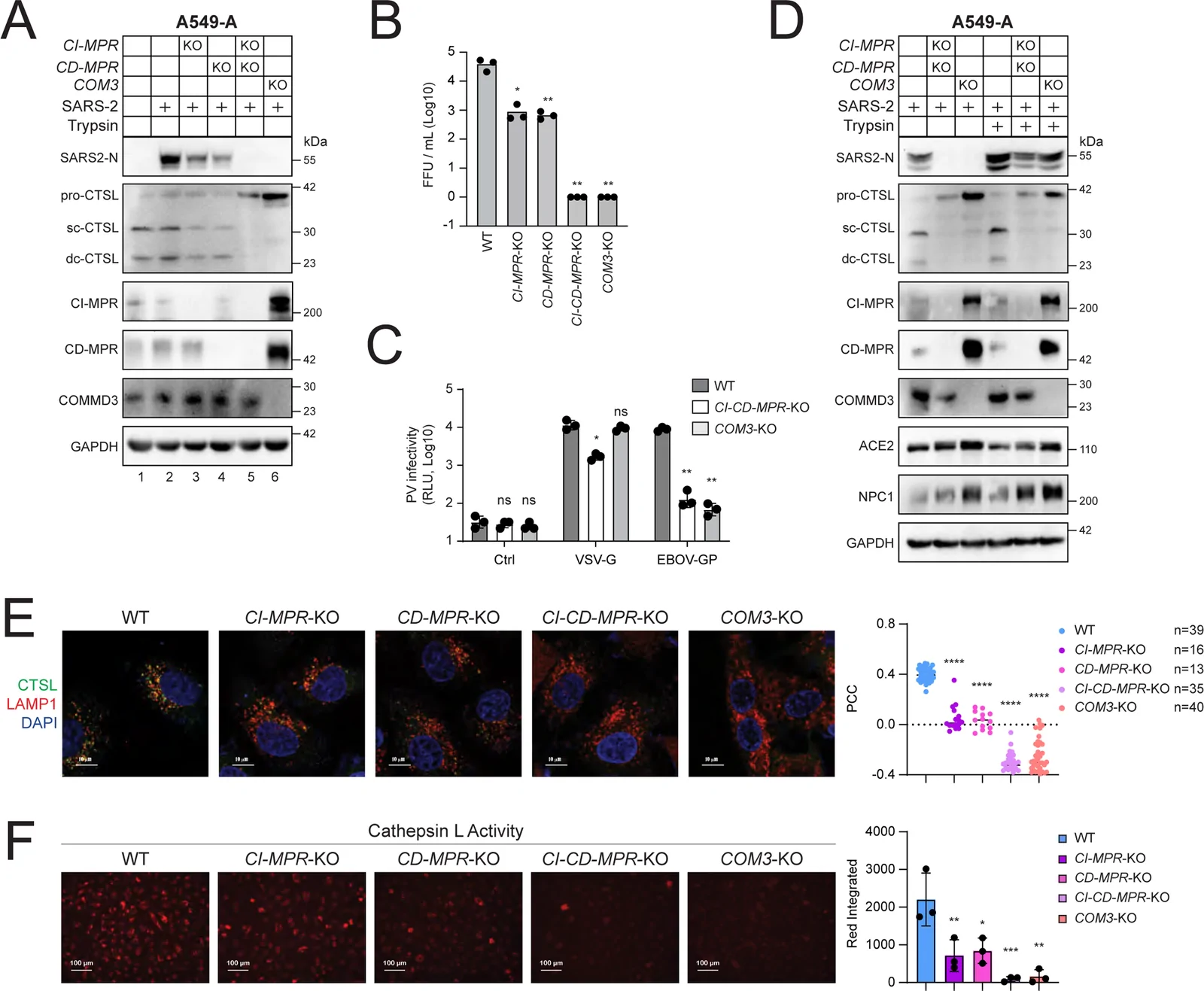
MPR-dependent trafficking links COMMD3 to CTSL maturation and viral entry. **A.** WT, CI-MPR-KO, CD-MPR-KO, CI-MPR/CD-MPR double-KO, and COM3-KO A549-A cells were infected with SARS2 WA1 at an MOI of 0.1 and harvested at 24 h post-infection. Western blot analysis of SARS2 N protein and CTSL expressions is shown. **B.** Viral titers in culture supernatants from WT, CI-MPR-KO, CD-MPR-KO, CI-MPR/CD-MPR double-KO, and COM3-KO A549-A cells were determined by FFU assay. Data are presented as mean ± SD. Each dot represents one biologically independent experiment (*n* = 3). One-way ANOVA followed by Dunnett’s multiple-comparison test using WT as the control. *P < 0.05; **P < 0.01. **C.** WT, CI-MPR/CD-MPR double-KO, and COM3-KO A549-A cells were infected with PVs bearing the indicated viral glycoproteins. Firefly luciferase activity was measured at 48 h post-infection. Data are presented as mean ± SD. Each dot represents one biologically independent experiment (*n* = 3). Two-way ANOVA followed by Dunnett’s multiple-comparison test using WT as the control. ns, not significant; *P < 0.05; **P < 0.01. **D.** WT, COM3-KO, and rescued A549-A cells were infected with SARS2 WA1 at an MOI of 0.1 in the presence or absence of trypsin and harvested at 24 h post-infection. Western blot analysis of SARS2 N protein, CTSL, ACE2 and NPC1 expression is shown. **E.** Representative immunofluorescence images of WT, CI-MPR-KO, CD-MPR-KO, CI-MPR/CD-MPR double-KO, and COM3-KO A549-A cells. Green, CTSL; red, LAMP1; blue, DAPI. Quantification of PCC between CTSL and LAMP1 is shown at right. PCC was calculated using an intensity threshold of 0.05. Each dot represents one field of view. Sample sizes are indicated in the figure. One-way ANOVA followed by Dunnett’s multiple-comparison test using WT as the control. ****P < 0.0001. **F.** Representative fluorescence images showing CTSL activity in WT, CI-MPR-KO, CD-MPR-KO, CI-MPR/CD-MPR double-KO, and COM3-KO A549-A cells. Cells were incubated with the Magic Red CTSL substrate for 30 min before imaging. Quantification of integrated Magic Red fluorescence intensity in the cells is shown at right. Integrated fluorescence intensity was measured from pixels above a fixed threshold (80–255) applied identically to all images. Data are presented as mean ± SD. Each dot represents one biologically independent experiment (*n* = 3). One-way ANOVA followed by Dunnett’s multiple-comparison test using WT as the control. *P < 0.05; **P < 0.01; ***P < 0.001.

To determine whether the defects in viral entry and CTSL maturation caused by COMMD3 deficiency result from impaired MPR function, we generated *CI-MPR*-KO, *CD-MPR*-KO and *CI-MPR*/*CD-MPR* double-KO A549-A cells (**Fig. S1**, middle and bottom panels). Following infection with SARS-CoV-2 WA1, deletion of either MPR individually caused only a partial reduction in infection, whereas simultaneous deletion of CI-MPR and CD-MPR nearly abolished viral infection, as determined by viral protein expression (Fig. 5A) and focus-forming unit (FFU) assays (Fig. 5B). Thus, the two MPRs have partially redundant functions in supporting SARS-CoV-2 infection, with their combined loss phenocopying COMMD3 deficiency. Consistent with this finding, CI-MPR/CD-MPR double knockout also abolished EBOV-GP-mediated pseudovirus entry while leaving VSV-G-mediated entry largely unaffected (Fig. 5C), recapitulating the selective viral entry phenotype observed following COMMD3 loss.

We next asked whether this entry defect resulted from insufficient proteolytic activation of the viral glycoprotein. SARS-CoV-2 WA1 virions were treated with trypsin before infection to bypass the requirement for endogenous endolysosomal protease-mediated Spike activation. Trypsin treatment restored SARS-CoV-2 infection in both *COM3*-KO and *CI-MPR*/*CD-MPR* double-KO cells (Fig. 5D). These findings place the entry defect downstream of receptor engagement and support defective proteolytic activation of Spike as the basis for impaired SARS-CoV-2 entry in COMMD3- and MPR-deficient cells.

We therefore examined whether MPR loss recapitulated the CTSL maturation defect caused by COMMD3 deficiency. Deletion of either CI-MPR or CD-MPR individually partially impaired CTSL maturation, whereas combined deletion of both receptors nearly abolished the generation of mature CTSL, closely phenocopying *COM3*-KO cells (Fig. 5A). Immunofluorescence microscopy similarly revealed a partial loss of mature lysosomal CTSL following deletion of either receptor and a pronounced loss in the double-KO cells (Fig. 5E). Consistent with these biochemical and imaging results, intracellular CTSL activity was partially reduced in each single-KO line and nearly abolished following deletion of both MPRs (Fig. 5F).

Together, these results establish a functional link between COMMD3, MPR-dependent trafficking and CTSL maturation. CI-MPR and CD-MPR act redundantly to sustain the generation of active CTSL, and their combined loss recapitulates the defects in CTSL maturation and CTSL-dependent viral entry caused by COMMD3 deficiency. These findings support a model in which COMMD3 promotes viral entry by maintaining MPR trafficking required for functional lysosomal CTSL. We next sought to define the region of COMMD3 responsible for this trafficking activity.

### The N-terminal domain of COMMD3 mediates MPR trafficking.

**Fig. 6.**
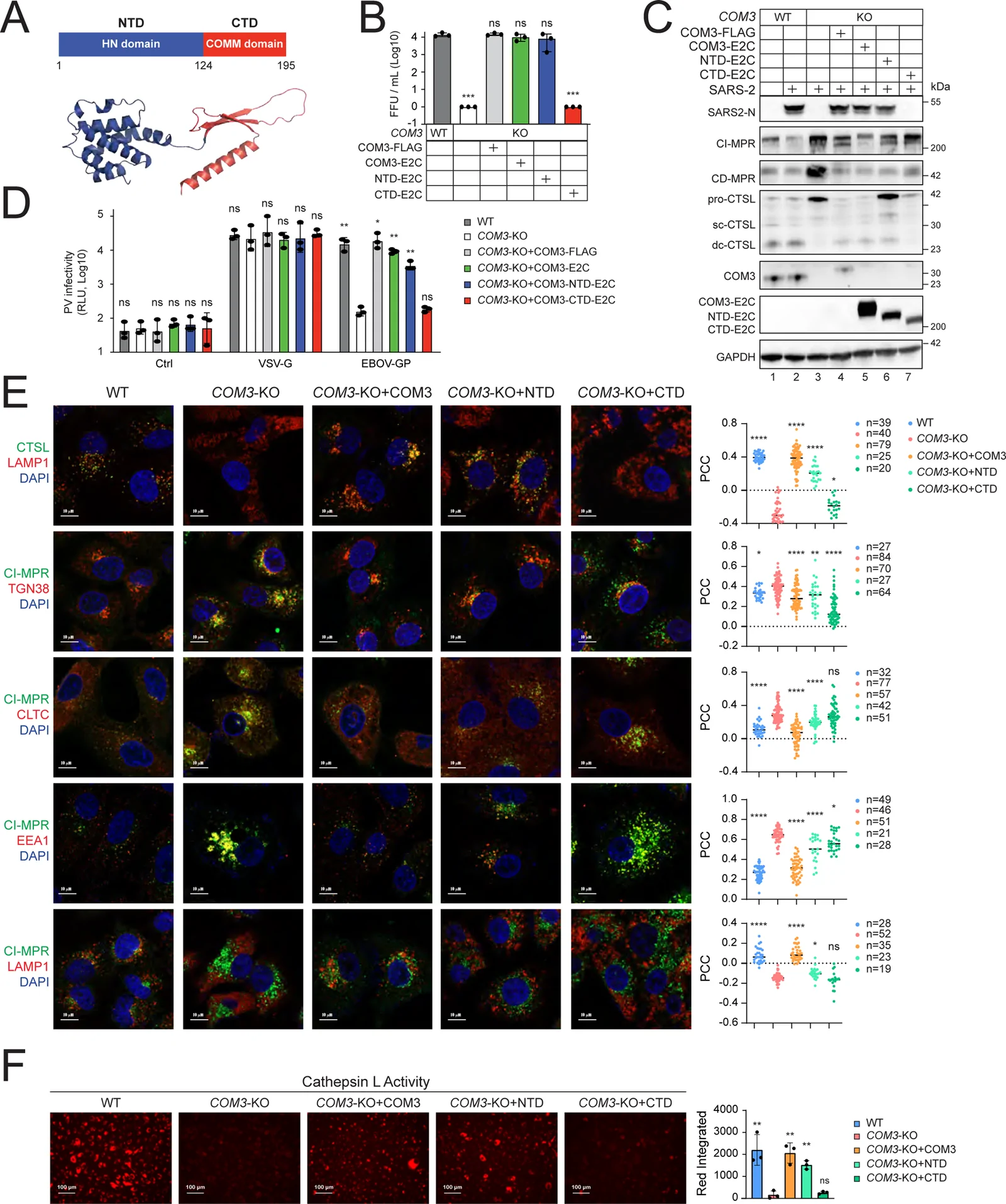
The N-terminal domain of COMMD3 mediates MPR trafficking. **A.** Schematic of the COMMD3 protein and its domain organization. **B.** WT, COM3-KO, and COM3-KO A549-A cells rescued with FLAG-tagged full-length COMMD3 (COM3-FLAG), E2-Crimson (E2C)-tagged full-length COMMD3 (COM3-E2C), E2C-tagged N-terminal COMMD3 (NTD-E2C), or E2C-tagged C-terminal COMMD3 (CTD-E2C) were infected with SARS2 WA1 at an MOI of 0.1 and harvested at 24 h post-infection. Viral titers in culture supernatants were determined by immunostaining-based FFU assay. Data are presented as mean ± SD. Each dot represents one biologically independent experiment (*n* = 3). One-way ANOVA followed by Dunnett’s multiple-comparison test using the WT group as the control. ns, not significant; ***P < 0.001. **C.** Western blot analysis of SARS2-infected cells in Fig. 6B. **D.** Indicated cells from Fig. 6B were infected with PVs bearing the indicated viral glycoproteins. Firefly luciferase activity was measured at 48 h post-infection. Data are presented as mean ± SD. Each dot represents one biologically independent experiment (*n* = 3). Two-way ANOVA followed by Dunnett’s multiple-comparison test using the COM3-KO group as the control. ns, not significant; *P < 0.05; **P < 0.01. **E.** Representative immunofluorescence images of indicated from Fig. 6B. Green, CTSL or CI-MPR; red, the indicated organelle markers; blue, DAPI. Quantification of Pearson’s correlation coefficient (PCC) between CTSL or CI-MPR and the indicated organelle markers is shown at right. PCC was calculated using an intensity threshold of 0.05. Each dot represents one field of view. Sample sizes are indicated in the figure. One-way ANOVA followed by Dunnett’s multiple-comparison test using the COM3-KO group as the control. ns, not significant; *P < 0.05; **P < 0.01; ****P < 0.0001. **F.** Representative fluorescence images showing CTSL activity in indicated cells from Fig. 6B. Cells were incubated with the Magic Red CTSL substrate for 30 min before imaging. Quantification of integrated Magic Red fluorescence intensity is shown at right. Integrated fluorescence intensity was measured from pixels above a fixed threshold (80–255) applied identically to all images. Data are presented as mean ± SD. Each dot represents one biologically independent experiment (*n* = 3). One-way ANOVA followed by Dunnett’s multiple-comparison test using the COM3-KO group as the control. ns, not significant; **P < 0.01.

To define the region of COMMD3 responsible for MPR trafficking, we generated constructs expressing either its N-terminal domain (NTD) or the conserved C-terminal COMM domain (CTD), which mediates assembly of COMMD proteins within the Commander complex (Fig. 6A). We stably expressed these constructs in *COM3*-KO cells and first assessed their ability to restore SARS-CoV-2 infection. Like full-length COMMD3, the NTD alone substantially rescued infection, restoring infectious progeny production to 3.75 log10 FFU/ml, approaching the 4.14 log10 FFU/ml observed in WT cells (Fig. 6B). By contrast, the isolated CTD failed to restore productive infection.

The ability of the COMMD3 domains to support viral infection correlated with their effects on MPR abundance and CTSL maturation. Expression of the NTD markedly reduced the accumulation of both CI-MPR and CD-MPR in *COM3*-KO cells and restored mature CTSL (Fig. 6C). The CTD produced only a partial reduction in MPR accumulation and was insufficient to restore CTSL maturation or SARS-CoV-2 infection. Consistent with these findings, the NTD also restored EBOV-GP-mediated entry (P = 0.0171), whereas the CTD showed no detectable rescue activity (Fig. 6D).

Immunofluorescence microscopy independently confirmed restoration of mature lysosomal CTSL by the NTD (Fig. 6E), which was accompanied by recovery of intracellular CTSL protease activity (Fig. 6F). Thus, the NTD is sufficient to restore both CTSL maturation and enzymatic function in COMMD3-deficient cells.

We next determined whether restoration of CTSL maturation by the NTD was associated with correction of the MPR trafficking defect. Relative to *COM3*-KO cells, NTD expression reduced CI-MPR localization within TGN, clathrin-associated and early endosomal compartments by 23%, 35% and 22%, respectively, while increasing its localization within late endosome/lysosome compartments by 11% (Fig. 6E). By contrast, although the CTD modestly reduced CI-MPR localization at the TGN, it did not significantly alter CI-MPR localization within clathrin-associated, early endosomal or late endosome/lysosome compartments. These findings indicate that the NTD is sufficient to promote CI-MPR progression from the compartments in which it accumulates following COMMD3 loss towards later endolysosomal compartments.

Together, these results identify the N-terminal domain of COMMD3 as the principal determinant of its MPR trafficking activity. The NTD is sufficient to correct MPR accumulation and trafficking, restore CTSL maturation and activity, and rescue CTSL-dependent viral entry, whereas the canonical COMM domain alone is insufficient. Thus, COMMD3 regulates the MPR–CTSL pathway primarily through its N-terminal region, revealing a function that is separable from the canonical COMM domain that mediates Commander complex assembly.

## Discussion

Our findings identify COMMD3 as a previously unrecognized regulator of MPR trafficking that links endosomal sorting to lysosomal protease maturation and function. Loss of COMMD3 caused marked accumulation of both CI-MPR and CD-MPR and redistributed these receptors towards early endosomal compartments, accompanied by profound impairment of CTSL maturation and activity. Combined deletion of CI-MPR and CD-MPR recapitulated the CTSL maturation and viral entry defects caused by COMMD3 loss, establishing a functional COMMD3-MPR-CTSL axis.

**Fig. 7.**
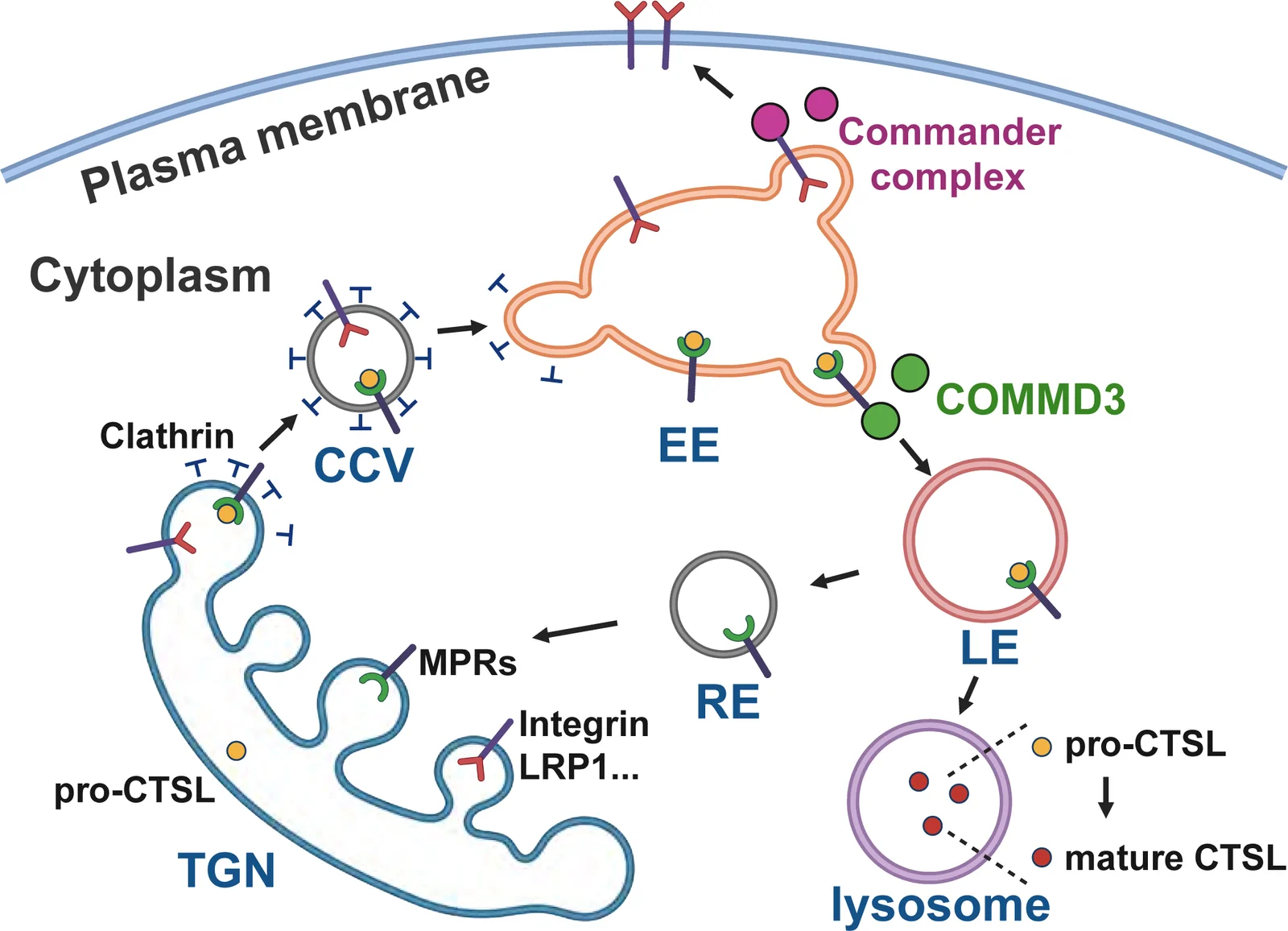
Schematic model of how COMMD3 promotes early endosomal exit of MPRs for lysosomal hydrolase delivery. CI-MPR and CD-MPR (collectively referred to as MPRs) bind newly synthesized lysosomal hydrolases, including pro-CTSL, in the trans-Golgi network (TGN) and transport them in clathrin-coated vesicles (CCVs) to early endosomes (EEs). COMMD3, through its N-terminal domain, promotes the exit of MPRs from EEs to late endosomes (LEs), where MPRs dissociate from their cargo, allowing pro-CTSL to be delivered to lysosomes and processed into mature CTSL. MPRs are subsequently sorted into recycling endosomes (REs) and returned to the TGN for additional rounds of lysosomal hydrolase transport. In contrast, the canonical Commander complex mediates the recycling of cell-surface receptors, such as integrins and LRP1, from endosomes back to the plasma membrane. Thus, COMMD3 coordinates MPR trafficking through a mechanism distinct from its established role in Commander complex assembly, thereby sustaining lysosomal hydrolase delivery, CTSL maturation, lysosome biogenesis, and endolysosomal homeostasis. Figure was created in BioRender.

Our results support a model in which COMMD3 promotes MPR exit from early endosomes and progression through the endosomal system (Fig. 7). COMMD3 associated with CD-MPR predominantly in early endosomes, whereas COMMD3 deficiency increased MPR localization within EEA1-positive and clathrin-associated compartments and reduced their distribution to later endosomal compartments. Notably, both MPRs accumulated markedly at the protein, but not transcript, level following COMMD3 deletion. Thus, increased MPR abundance does not reflect increased functional capacity; rather, their accumulation is associated with impaired CTSL maturation. One possibility is that MPRs trapped or delayed within early endosomes are unable to efficiently participate in repeated rounds of lysosomal hydrolase transport. How COMMD3 promotes MPR exit from early endosomes and how trafficking defects lead to receptor accumulation remain important questions for future investigation.

The consequences of defective MPR trafficking were particularly evident for CTSL. COMMD3 deficiency caused accumulation of pro-CTSL, profound loss of mature CTSL and markedly reduced CTSL activity. Combined deletion of both MPRs closely phenocopied these defects, whereas deletion of either receptor alone produced a partial phenotype, consistent with their partially redundant roles in lysosomal hydrolase delivery. Moreover, inhibition of endolysosomal acidification impaired CTSL maturation but did not fully reproduce the pronounced pro-CTSL accumulation caused by COMMD3 loss, suggesting that the phenotype extends beyond a simple defect in acidification-dependent proteolytic processing.

Unexpectedly, the MPR trafficking activity of COMMD3 mapped predominantly to its N-terminal domain (NTD), rather than the conserved C-terminal COMM domain that mediates Commander complex assembly. The NTD alone substantially corrected MPR trafficking, restored CTSL maturation and activity, and rescued viral entry, whereas the COMM-containing C-terminal domain was insufficient. These findings reveal a function of COMMD3 that is separable at the domain level from its canonical role within Commander and raise the possibility that the divergent N-terminal regions of other COMMD proteins confer additional cargo- or pathway-specific trafficking activities. Whether the COMMD3 NTD interacts directly with MPRs or recruits additional trafficking machinery remains to be determined.

Finally, our findings establish a functional connection between COMMD3-dependent MPR trafficking and viral entry. COMMD3 deficiency selectively impaired endosomal SARS-CoV-2 infection and EBOV-GP-mediated entry, while TMPRSS2 expression bypassed the requirement for COMMD3 during SARS-CoV-2 infection. Combined MPR deletion reproduced this phenotype, and exogenous proteolytic activation with trypsin restored SARS-CoV-2 infection in both COMMD3- and MPR-deficient cells. Thus, the viral entry defect reflects impaired endolysosomal proteolytic activation rather than altered receptor expression or an intrinsic defect in membrane fusion.

Together, our study identifies COMMD3 as a regulator of MPR trafficking and establishes a mechanistic link between endosomal sorting, CTSL maturation and virus entry. By promoting MPR progression from early endosomes, COMMD3 sustains functional lysosomal CTSL and thereby supports viruses that depend on endolysosomal proteolytic activation. The unexpected contribution of the COMMD3 NTD further suggests that COMMD proteins may possess specialized trafficking activities beyond their canonical functions within the Commander complex.

## Materials and Methods

### Cell culture.

Human alveolar epithelial A549 cells (BEI Resources, NR-52268), A549 cells stably expressing human ACE2 (A549-A; BEI Resources, NR-53821), human embryonic kidney HEK293T cells (ATCC, CRL-3216), African green monkey kidney Vero cells (ATCC, CRL-1586), and Vero cells stably expressing human ACE2 and TMPRSS2 (Vero-AT; BEI Resources, NR-54970) were maintained in Dulbecco’s modified Eagle’s medium (DMEM; Thermo Fisher Scientific, 11965084) supplemented with 10% fetal bovine serum (FBS; Biowest, S1620) and 1% penicillin-streptomycin (Thermo Fisher Scientific, 15140122). Cells were cultured at 37 °C in a humidified incubator with 5% CO_2_.

### Antibodies.

Primary antibodies used in this study included rabbit polyclonal antibodies against COMMD3 (Proteintech, 26240–1-AP), CI-MPR (Proteintech, 20253–1-AP), EEA1 (Proteintech, 28347–1-AP), RAB7A (Proteintech, 55469–1-AP), cathepsin L (CTSL; Proteintech, 27952–1-AP), NPC1 (Proteintech, 13926–1-AP) and TMPRSS2 (Proteintech, 14437–1-AP); rabbit monoclonal antibodies against HA (Proteintech, HRP-81290), FLAG (Proteintech, HRP-80801–2) and SARS-CoV-2 nucleocapsid (Sino Biological, 40143-R019); and guinea pig polyclonal anti-SARS-CoV-2 antibody (BEI Resources, NR-10361; 1:15,000). Mouse monoclonal antibodies included those against CD-MPR (Santa Cruz Biotechnology, sc-365196), EEA1 (Proteintech, 68065–1-Ig), TGN46 (Santa Cruz Biotechnology, sc-166594), clathrin heavy chain (CLTC; Proteintech, 66487–1-Ig), LAMP1 (Cell Signaling Technology, 15665), ACE2 (Proteintech, 66699–1-Ig) and GAPDH (Proteintech, HRP-60004).

Secondary antibodies included horseradish peroxidase (HRP)-conjugated goat anti-rabbit IgG (H+L) (Proteintech, SA00001–2), HRP-conjugated goat anti-mouse IgG (H+L) (Proteintech, CA00001–1), HRP-conjugated goat anti-guinea pig IgG (Thermo Fisher Scientific, A16104), Alexa Fluor 488-conjugated goat anti-rabbit IgG (H+L) (Thermo Fisher Scientific, A-11008) and CoraLite Plus 594-conjugated goat anti-mouse IgG (H+L) (Proteintech, RGAM004).

Unless otherwise indicated, antibodies were used at the dilutions recommended by the manufacturers.

### Chemicals.

Reagents used in this study included bafilomycin A1 (MedChemExpress, HY-100558), concanamycin A (MedChemExpress, HY-N1724), TPCK-treated trypsin (Sigma-Aldrich, T1426), Lipofectamine 3000 Transfection Reagent (Thermo Fisher Scientific, L3000015), puromycin (InvivoGen, ant-pr-1), blasticidin (InvivoGen, ant-bl-05), G418 sulfate (InvivoGen, ant-gn-1), zeocin (InvivoGen, ant-zn-05), and hygromycin B (MedChemExpress, HY-B0494).

### Plasmid vectors.

The following plasmids were obtained from Addgene: lentiCRISPRv2 puro (#98290), lentiCRISPRv2 blast (#98293), lentiCRISPRv2 neo (#98292), pCMV-VSV-G (#8454), p8.91 (#187441), pEBB-COMMD3-FLAG (#74895), pBa.M6PR-GFP (#224407), hCathepsin L (#11250), pcDNA3.1-hACE2-LgBiT (#215837), pLenti-CMV-TMPRSS2-TwinStrep-Zeo (#192000), HA-Rab7A (#131417), Lamp1-RFP (#1817), mRFP-Rab5 (#14437), mCherry-Rab7A (#61804), DsRed-Rab11 WT (#12679), mCherry-TGNP-N-10 (#55145), CD63-pEGFP-C2 (#62964), and pEF.myc.ER-E2-Crimson (#38770).

The plasmids pNL4.3-ΔEnv-Luc, pcDNA3.1-EBOV-GPΔMLD, pCAGGS-SARS-CoV-2-S-ΔC19-FLAG, and pLenti-BSD-hTMPRSS2-FLAG were described previously^16^. pcDNA3.1-NefSF2-VN-HA and pcDNA3.1-mSer5-FLAG-VC were described previously^20^. pMSCVhyg and pcGP were described previously^21^. pcDNA3.1 vectors expressing ERManI (MAN1B1) and its catalytic mutant E330A were described previously^18, 22^. Plasmids expressing influenza A virus A/Thailand/1(KAN-1)/2004 (H5N1) HA and NA were described previously^23^.

For CRISPR-Cas9-mediated gene knockout, the COMMD3 sgRNA (CTTGAAACATATCGACCCAG) was cloned into lentiCRISPRv2-puro and lentiCRISPRv2-blast, the CI-MPR sgRNA (GCTCAAAGATCCATTCGCCG) into lentiCRISPRv2-neo, and the CD-MPR sgRNA (GAAAAAACTTGCGACTTGGT) into lentiCRISPRv2-puro using BsmBI restriction sites, generating lentiCRISPRv2-sgCOMMD3-puro, lentiCRISPRv2-sgCOMMD3-blast, lentiCRISPRv2-sgCI-MPR-neo, and lentiCRISPRv2-sgCD-MPR-puro.

For ectopic expression, the COMMD3 coding sequence from pEBB-COMMD3-FLAG was subcloned into pMSCVhyg via BglII/XhoI digestion to generate pMSCVhyg-COMMD3-FLAG. Full-length COMMD3, the N-terminal domain (NTD; amino acids 1–124), and the C-terminal domain (CTD; amino acids 125–195) were fused to E2-Crimson and cloned into pMSCVhyg to generate pMSCVhyg-COMMD3-E2C, pMSCVhyg-NTD-E2C, and pMSCVhyg-CTD-E2C. mCherry-Rab7A was similarly cloned into pMSCVhyg to generate pMSCVhyg-mCherry-Rab7A.

The ACE2 coding sequence from pcDNA3.1-hACE2-LgBiT was cloned into pLenti-BSD-hTMPRSS2-FLAG via XbaI/BamHI digestion to generate pLenti-BSD-hACE2-FLAG. ACE2-FLAG and TMPRSS2-FLAG expression constructs were subsequently generated by subcloning the corresponding coding sequences into the HA-Rab7A vector via HindIII/XbaI digestion. ERManI and ERManI-E330A were also subcloned into the HA-Rab7A vector via BamHI/XhoI digestion.

For bimolecular fluorescence complementation (BiFC), COMMD3 was fused to the VN173 fragment from pcDNA3.1-NefSF2-VN-HA and cloned into the HA-RAB7A vector to generate HA-COMMD3-VN173. ACE2 was fused to the VC155 fragment from pcDNA3.1-mSer5-FLAG-VC to generate FLAG-ACE2-VC155. The VC155 sequence was also fused to hCathepsin L to generate FLAG-CTSL-VC155, and the CD-MPR coding sequence from pBa.M6PR-GFP was cloned into this vector to generate FLAG-CD-MPR-VC155.

RAB9A was amplified from cellular cDNA and cloned into the mCherry-RAB7A vector to generate mCherry-RAB9A. CD63 was subcloned from CD63-pEGFP-C2 into the mCherry-Rab7A vector to generate mCherry-CD63. All plasmids generated in this study were verified by Sanger sequencing.

### Transfection.

HEK293T cells were transfected with Lipofectamine 3000 (Thermo Fisher Scientific, L3000015) according to the manufacturer’s instructions. Briefly, plasmid DNA, P3000 reagent, and Lipofectamine 3000 were each diluted in Opti-MEM medium (Thermo Fisher Scientific, 31985062), combined, and incubated at room temperature for 10 min. The transfection mixture was then added to HEK293T cells at 70–90% confluence, and the culture medium was replaced 4 h later.

### Stable cell line production.

To generate CRISPR-Cas9 knockout cell lines, lentiCRISPRv2-sgCOMMD3-puro, lentiCRISPRv2-sgCOMMD3-blast, lentiCRISPRv2-sgCI-MPR-neo, or lentiCRISPRv2-sgCD-MPR-puro was co-transfected with p8.91 and pCMV-VSV-G into HEK293T cells at a plasmid ratio of 4:2:1 to produce lentiviral particles. Target cells were transduced with lentiviruses for 48 h and selected with the appropriate antibiotics. Single-cell clones were isolated by single-cell sorting into 96-well plates using a MoFlo Astrios EQ cell sorter and validated by Sanger sequencing and Western blotting. The following knockout cell lines were generated: COMMD3-KO A549 (puromycin), COMMD3-KO A549-A (puromycin), COMMD3-KO HEK293T (blasticidin), CI-MPR-KO A549-A (G418), CD-MPR-KO A549-A (puromycin), and CI-MPR/CD-MPR double-KO A549-A (G418 and puromycin).

To generate stable expression cell lines, pLenti-CMV-TMPRSS2-TwinStrep-Zeo was co-transfected with p8.91 and pCMV-VSV-G into HEK293T cells to produce lentiviral particles, whereas pMSCVhyg-COMMD3-FLAG, pMSCVhyg-COMMD3-E2C, pMSCVhyg-COMMD3-NTD-E2C, pMSCVhyg-COMMD3-CTD-E2C, or pMSCVhyg-mCherry-Rab7A was co-transfected with pcGP and pCMV-VSV-G to produce retroviral particles (plasmid ratio, 4:2:1). Target cells were transduced for 48 h, followed by antibiotic selection and validation by Western blotting. Stable cell lines generated in this study included COMMD3-FLAG-expressing COMMD3-KO A549 and A549-A cells; COMMD3-E2C-, COMMD3-NTD-E2C-, and COMMD3-CTD-E2C-expressing COMMD3-KO A549-A cells; TMPRSS2-expressing WT, COMMD3-KO, and COMMD3-KO/COMMD3-FLAG-complemented A549-A cells; and mCherry-RAB7A-expressing WT and COMMD3-KO A549-A cells.

### SARS-CoV-2 viruses.

The following SARS-CoV-2 strains were obtained through BEI Resources, NIAID, NIH: USA-WA1/2020 (WA1; NR-52281), icSARS-CoV-2-eGFP (NR-54002), hCoV-19/USA/MD-HP05285/2021 (B.1.617.2, Delta; NR-55671), and hCoV-19/USA/NY-MSHSPSP-PV56475/2022 (BA.2.12.1, Omicron; NR-56781). Virus stocks were propagated in Vero-AT cells and harvested at 48 h post-infection.

### SARS-CoV-2 focus-forming assay.

SARS-CoV-2 infection was performed in the biosafety level 2 facility at University of Illinois Chicago. Cells were infected with SARS-CoV-2 at a multiplicity of infection (MOI) of 0.1 for 1 hour, washed three times with PBS, and cultured for the indicated times. Cell lysates were collected for Western blot analysis of viral N protein expression, and supernatants were collected for viral titration using a previously described focus-forming assay^24^. Briefly, serial dilutions of culture supernatants were added to confluent monolayers of Vero E6 cells seeded in 96-well plates. After 1 hour of incubation, the cells were overlaid with 1% (wt/vol) methylcellulose in minimal essential medium (MEM) supplemented with 2% FBS. At 24 hours post-infection, the cells were fixed with 4% paraformaldehyde (PFA) for 15 min. Foci were detected by immunostaining with a guinea pig polyclonal anti-SARS-CoV-2 primary antibody (BEI Resources, NR-10361; 1:15,000), followed by a horseradish peroxidase-conjugated goat anti-guinea pig secondary antibody (Thermo Fisher Scientific, A16104; 1:5,000) diluted in PermWash buffer (PBS containing 0.1% saponin and 0.1% bovine serum albumin). Following TrueBlue peroxidase substrate treatment (SeraCare, 5510–0030), focus-forming units (FFU) were quantified using an ImmunoSpot ELISpot plate scanner (Cellular Technology Limited).

### HIV-1-based pseudoviruses (PVs) production and infection.

HEK293T cells were cultured in 10-cm dishes and co-transfected with 10 μg of pNL4.3-ΔEnv-Luc and 2.5 μg of the indicated viral glycoprotein expression plasmid (pcDNA3.1-EBOV-GPΔMLD, pCAGGS-SARS2-S-ΔC19-FLAG, 7705-HA/7708-NA). Supernatants were harvested at 48 hours post-transfection and centrifuged twice at 5,000 × g for 10 min to remove cell debris, passed through a 0.22-μm filter, and stored at −80 °C until use. PVs were normalized by p24^Gag^ ELISA, and equal amounts of viruses were used to infect A549-A cells. After 48 h, firefly luciferase activities were determined using Firefly Luciferase Assay Kit (Biotium, 30085).

### Transmission electron microscopy (TEM).

Cells grown in culture dishes were fixed with 2.5% glutaraldehyde in 0.1 M sodium phosphate buffer (pH 7.2–7.4) for 1 hour at room temperature and then stored in the same fixative at 4 °C until further processing. Samples were processed, embedded, sectioned, stained, and imaged by the Electron Microscopy Core in Research Resources Center of University of Illinois at Chicago according to standard procedures.

### Western blot.

Cells were collected and washed twice with PBS and lysed with cell lysis buffer (Cell Signaling Technology, 9803) containing a protease inhibitor cocktail (Sigma-Aldrich, P8340). Cell lysate was centrifuged at 12,000 × g for 10 min at 4°C, and the supernatants were collected and mixed with Laemmli Sample Buffer (BIO-RAD, 1610747). Equal amounts of protein were separated by SDS-polyacrylamide gel electrophoresis (SDS-PAGE) and transferred to PVDF membranes. Membranes were probed with the indicated primary and secondary antibodies, and signals were detected using Western HRP substrate (Millipore, WBLUC0100).

### Reverse transcription and quantitative real-time PCR (RT-qPCR).

A two-step RT-qPCR was used to examine specific mRNA levels. Total RNA was extracted and purified by using TRIzol Reagent (Thermo Fisher Scientific, 15596026). Reverse transcription was performed using One-step RT-gDNA Digestion SuperMix for qPCR (Yeasen, 11155ES). qPCR was performed using Hieff UNICON Universal Blue qPCR Master Mix (SYBR) (Yeasen, 11184ES). The primer sequences were as follows: GAPDH, forward 5′-GTCTCCTCTGACTTCAACAGCG-3′ and reverse 5′-ACCACCCTGTTGCTGTAGCCAA-3′; CI-MPR, forward 5′-CAGAGCGGAGGTTCATCCTAT-3′ and reverse 5′-CGAATATCGGAGGGTCTGATTGT-3′; CD-MPR, forward 5′-ACTTGCGACTTGGTAGGAGAA-3′ and reverse 5′-CCGGCACACCCTGAAGATG-3′; CTSL, forward 5′-AAACTGGGAGGCTTATCTCACT-3′ and reverse 5′-GCATAATCCATTAGGCCACCA-3′. Relative gene expression was normalized to GAPDH and calculated using the 2^−ΔΔCt^ method.

### Immunofluorescence.

Cells grown on coverslips were fixed with 4% paraformaldehyde (PFA) for 15 min, permeabilized with 0.1% Triton X-100 in PBS for 10 min and blocked with 5% bovine serum albumin (BSA) for 1 hour at room temperature. Cells were then incubated with primary antibodies overnight at 4 °C, followed by fluorescent secondary antibodies for 1 hour at room temperature. Nuclei were stained with DAPI. Images were acquired using a Zeiss LSM 980 confocal microscope. Images were analyzed using R (version 4.6.0). Integrated fluorescence intensities and Pearson’s correlation coefficients (PCCs) were calculated using an intensity threshold of 0.05.

### Flow cytometry.

Cells were harvested, washed with PBS, and resuspended in PBS containing 2% FBS. For surface ACE2 detection, cells were incubated with an anti-ACE2 antibody for 30 min at 4 °C, followed by a fluorescent secondary antibody for 30 min at 4 °C in the dark. Data acquisition on a CytoFLEX S flow cytometer was performed by the Flow Cytometry Core in Research Resources Center of University of Illinois at Chicago. Data were analyzed using FlowJo (BD Biosciences).

### Bimolecular fluorescence complementation (BiFC) assays.

FLAG-ACE2-VC155, FLAG-CTSL-VC155, and FLAG-CD-MPR-VC155 were individually co-transfected with HA-COMMD3-VN173 into HEK293T cells using Lipofectamine 3000 according to the manufacturer’s instructions. At 36 hours post-transfection, cells were collected. To verify the expression of BiFC fusion proteins, Western blotting was performed to detect VN-tagged proteins using an anti-HA antibody and VC-tagged proteins using an anti-FLAG antibody. To quantify BiFC fluorescence, flow cytometry was performed to determine the percentage of positive cells using the 488-nm laser. To assess the co-localization of COMMD3 and CD-MPR BiFC fluorescence with different organelles, FLAG-CD-MPR-VC155, HA-COMMD3-VN173, and each individual organelle marker plasmid (RAB5A, RAB7A, RAB9A, RAB11A, LAMP1, TGN46, or CD63) were co-transfected into HEK293T cells. At 36 hours post-transfection, cells were fixed with 4% PFA for 15 min and permeabilized with 0.1% Triton X-100 in PBS for 10 min at room temperature. Nuclei were stained with DAPI. Cells were imaged using a Zeiss LSM 980 confocal microscope. Images were analyzed using R. PCCs were calculated using an intensity threshold of 0.05.

### Percoll gradient fractionation.

Subcellular fractionation by Percoll gradient centrifugation was performed as previously described ^25^. Briefly, A549-A cells were homogenized in homogenization buffer (250 mM sucrose, 20 mM Tris-HCl, pH 7.4, 1 mM EGTA, 1 mM EDTA, and protease inhibitor cocktail). Unbroken cells and nuclei were removed by centrifugation at 750 × g for 10 min at 4 °C. The supernatant was layered onto nine volumes of 20% (vol/vol) Percoll and centrifuged at 20,000 × g for 2.5 h at 4 °C. Twelve fractions were sequentially collected from top to bottom, and equal amounts of each fraction were analyzed by western blot with the indicated antibodies. The distribution of subcellular compartments was determined using the indicated organelle markers.

### Cathepsin L activity assay.

Cathepsin L (CTSL) activity was assessed using the Magic Red Cathepsin L Kit (Bio-Rad, ICT941) according to the manufacturer’s instructions. Cells were incubated with the Magic Red CTSL substrate for 30 min at 37°C and subsequently subjected to fluorescence imaging. CTSL activity was quantified by measuring the integrated fluorescence intensity of Magic Red using a fixed threshold (80–255) applied identically to all images.

### LC–MS/MS-based proteomic analysis.

Wild-type and COM3-KO A549-A cells, with three independent biological replicates per group, were subjected to proteomic analysis. Cells were lysed in cell lysis buffer supplemented with protease inhibitors. For each sample, 20 μg of protein was processed using a filter-aided sample preparation (FASP) protocol. Proteins were reduced with dithiothreitol (DTT), alkylated with iodoacetamide (IAA), and digested with trypsin overnight at 37°C. The resulting peptides were desalted and subjected to LC–MS/MS analysis using a Q Exactive HF mass spectrometer coupled to an UltiMate 3000 RSLC system. MS/MS spectra were analyzed using MaxQuant (v2.0.3.0), with the false discovery rate (FDR) controlled at 1% for peptide and protein identification. Label-free quantification (LFQ) was used for protein quantification. The analysis was performed by the Mass Spectrometry Core in Research Resources Center of University of Illinois at Chicago. Subsequent data analysis and visualization were performed using R (version 4.6.0). The mass spectrometry proteomics data have been deposited to the ProteomeXchange Consortium (https://proteomecentral.proteomexchange.org) via the iProX partner repository^26, 27^ with the dataset identifier PXD081590.

### Statistical analysis.

Quantitative data are presented as mean ± standard deviation (SD) from at least three independent experiments, unless otherwise indicated in the figure legends. Statistical analyses were performed using GraphPad Prism. Statistical comparisons between two groups were performed using a two-tailed Welch’s t-test. Comparisons among multiple groups were performed using one-way ANOVA followed by Dunnett’s multiple-comparisons test. Two-way ANOVA was used for experiments involving two independent factors, followed by Dunnett’s multiple-comparisons test. Levels of significance were indicated as: ns, not significant; *P < 0.05; **P < 0.01; ***P < 0.001; ****P < 0.0001.

## Supplementary Material

Supplementary Files

This is a list of supplementary files associated with this preprint. Click to download.

• TableS1.xlsx

• TableS1.xlsx

• TableS1.xlsx

• FiguresandSupplementalFigurescompressed.pdf

• FiguresandSupplementalFigurescompressed.pdf

• FiguresandSupplementalFigurescompressed.pdf

Table S1. Proteomic analysis of wild-type and COM3-KO A549-A cells by Mass Spectrometry.

Figure S1 to S7.

## Data Availability

Source data are provided with this paper.
